# Supplementary material for: Femtoliter Batch Reactors for Nanofluidic Scattering Spectroscopy Analysis of Catalytic Reactions on Single Nanoparticles
Source: Small Methods. 2025 Jun 4;10(2):2500693. doi: 10.1002/smtd.202500693 (PMC12825324; doi:10.1002/smtd.202500693)
Supplement: Supplementary file 1 — Supporting Information [file SMTD-10-2500693-s001.docx]

**Supplementary Material for**

Femtoliter Batch Reactors for Nanofluidic Scattering Spectroscopy Analysis of Catalytic Reactions on Single Nanoparticles

*Björn Altenburger^1^, Joachim Fritzsche^1^ and Christoph Langhammer^1*^*

^1^Department of Physics, Chalmers University of Technology; SE-412 96 Gothenburg, Sweden

*Corresponding author: clangham@chalmers.se


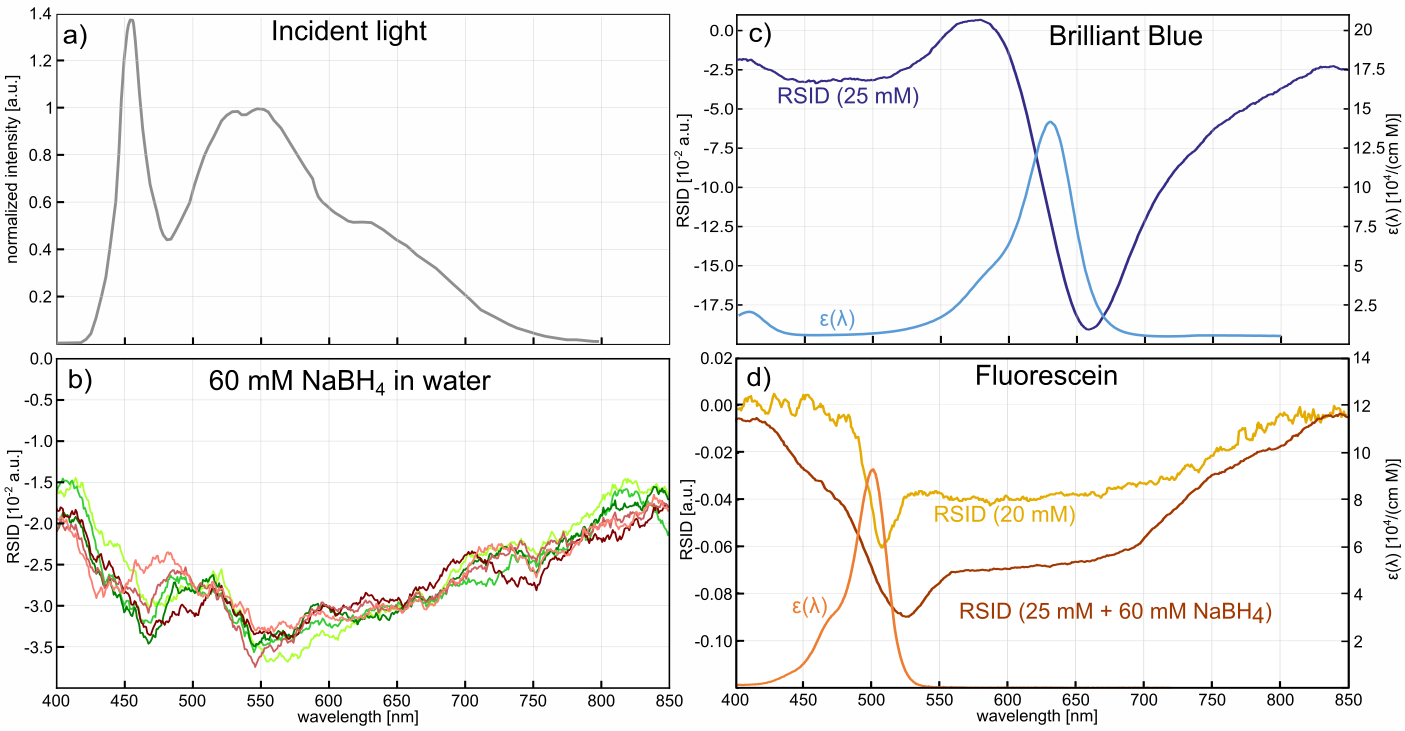


**Figure S1. *Collection of spectra relevant for this work.*** *a) Emission intensity spectrum of the light source (Thorlabs Solis 3C) used for the NSS measurements. b) RSID spectrum of a 60 mM NaBH4 solution measured in a nanochannel. The color code depicts the six nanochannel sections used for NSS readout and is the same as introduced in* ***Figure 3*** *in the main text. c) RSID spectrum of a 25 mM aqueous Brilliant Blue solution together with the molar extinction coefficient spectrum,* $\varepsilon(\lambda)$*, calculated from the RSID spectrum using the formalism we have introduced earlier.*^[1]^ *We note that the main peak of the* $\varepsilon(\lambda)$ *spectrum corresponds to the negative slope of the RSID spectrum. d) RSID spectra of a 20 mM and 25 mM Fluorescein solution, where the latter has been mixed with 60 mM NaBH_4_ to increase the pH value. We note the higher amplitude of the negative RSID peak in alkaline environment compared to the one in just MilliQ water, as well as the distinct shift to longer wavelengths at the higher pH. Also plotted is a Fluorescein* $\varepsilon(\lambda)$ *spectrum measured using a spectrophotometer. We again note that the* $\varepsilon(\lambda)$ *peak maximum is localized at the negative slope of the negative RSID peak of the aqueous fluorescein solution, as expected.*


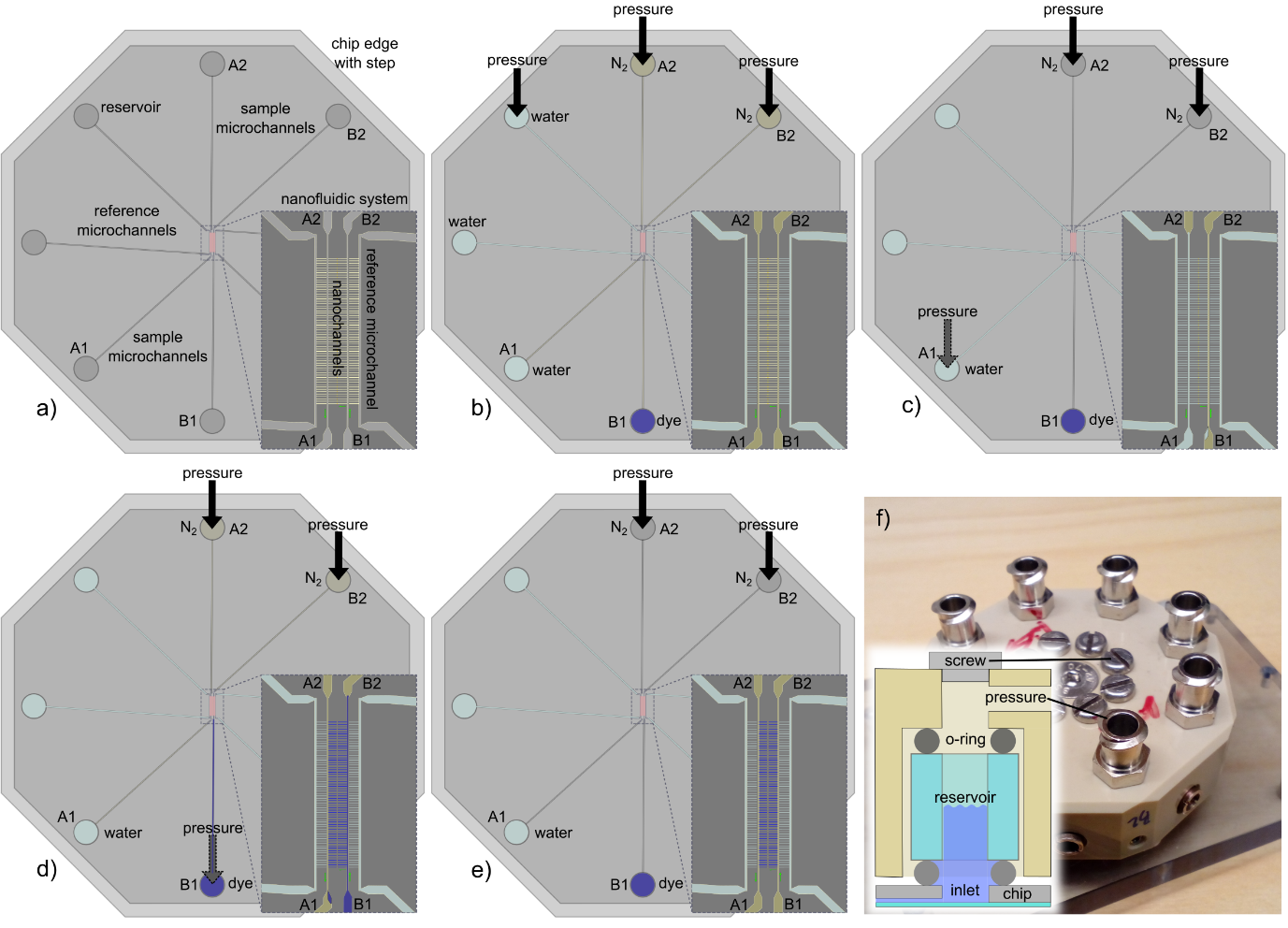


**Figure S2. *Micro and nanofluidic operation scheme.*** *a) The fluidic chip is a silicon-based platform that comprises micro- and nanochannels, where the former connect to inlet reservoirs on one end and to the nanochannel system in the center (inset) on the other end. After fabrication, the entire fluidic system is filled with air. b) When implementing the batch reactor according to the steps summarized in* ***Figure 4a-d*** *in the main text, to execute the first step, the reservoirs of the reference microchannels are filled with water and pressurized with N_2_ gas to create a flow of water through the reference microchannels to fill the reference nanochannels with water (see inset). At the same time, inlet A1 is filled with water and inlet B1 is filled with the BB-dye solution. To keep the liquids in inlets A1 and B1 from entering the sample nanochannel system too early, N_2_ gas is flushed in through inlets A2 and B2 by applying a pressure of 1500 mbar. c) For the second step, measuring the intrinsic difference spectra with NSS, the central nanochannels are flushed with water (****cf.*** ***Figure 4b****). To do so, pressure is applied to the inlet reservoir A1 with a syringe, thereby counteracting the still applied N_2_ pressure in the microchannel until the water reaches the nanochannels. The overpressure on the water side and the capillary forces causes the water to flush through the nanochannels (see inset) and then be carried away by the N_2_ gas flow on the exit side. d) To flush the dye solution into the nanochannels in the third step, (****cf. Figure 4c****), the pressure on A1 is released and instead applied to B1. It is here of importance to limit the extension of the dye solution to the thinner sections of the microchannel (as shown in the inset), as an extension into the microchannel towards B2 could cause solution pockets that disturb the concentration in the nanochannels later. It is also recommended that the nanochannels are flushed thoroughly with the dye solution before the next step, as the water that has been flushed through to the dye side and has remained in the microchannel toward B1 changes the concentration of the first part of the dye solution when it is flushed in. e) To close off the nanochannels and establish the batch reactor condition in the fourth step (****cf. Figure 4d****), the pressure on B1 is released. The N_2_ pressure on B2 pushes the liquid out of the thinner sections of the microchannel while the nanochannels remain filled with the dye solution (inset). If it is of interest to repeat the procedure and fil a batch reactor once again, it is necessary to exchange the liquids in reservoirs A1 and B1 since they are contaminated. f) The octagonal chips are clamped in a holder that provides a short glass tube for each inlet to function as reservoirs. These tubes are tightened to the chip via O-rings (see inset). Liquid can be inserted via a screw over the reservoirs while pressure is applied via side connections and Luer-Lock couplings, using a Fluidgent device or a syringe.*


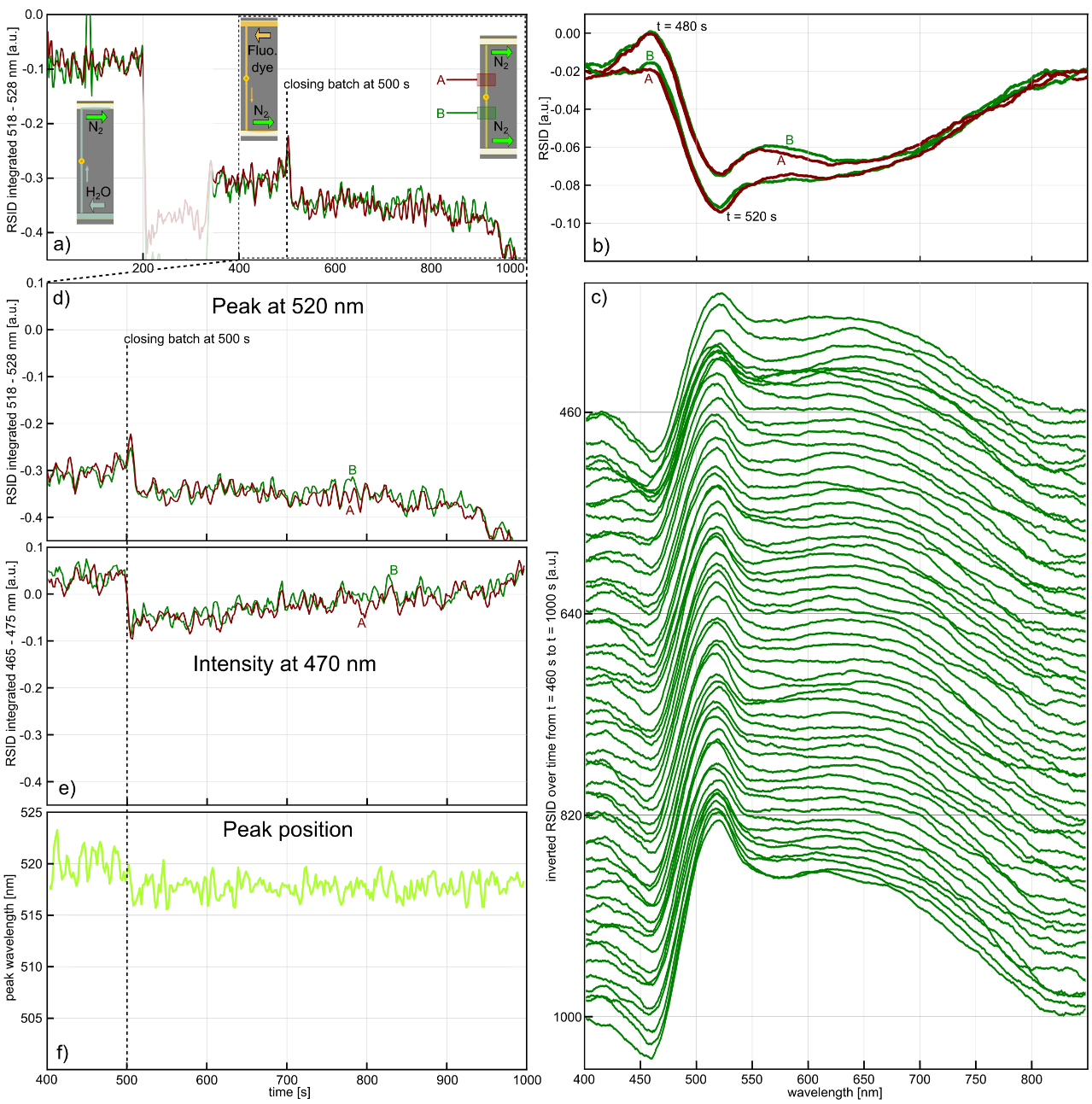


**Figure S3. *Batch reactor experiment with empty nanochannel without Au nanoparticle.*** *a) Time trace of the integrated RSID value between 518 – 528 nm for the same two nanochannel sections as depicted in* ***Figure 5*** *in the main text. The same three subsequent steps in the batch reactor operation are indicated along the time trace: i) flushing of water (0 – 200 s) flushing of the 25 mM fluorescein and 120 mM NaBH_4_ solution (350 – 500 s), closed batch reactor (500 – 1000 s). The shaded area marks the time where monitoring of the sample through the microscope eyepiece was necessary. b) RSID spectra for the two sections of the nanochannel at a time before (480 s) and after (540 s) the batch reactor was closed at 500 s. c) Ridge plot of the inverted RSID spectra taken at 10 s intervals from t = 460 s to 1000 s. In contrast to* ***Figure 5c*** *in the main text, where the Au nanoparticle is present in the nanochannel,* *no significant change of the RSID spectrum occurs and no new short-wavelength peak emerges. d) Zoom-in of the time trace depicted in a) that follows the integrated amplitude of the main RSID peak, that corresponds to the fluorescein absorption band in alkaline environment. We attribute the slight increase of negative peak amplitude towards more negative values in the final stages of the experiment to the onset of channel drying, as discussed in* ***Figure 4*** *in the main text. e) Same as d) but when integrating the RISD between 465 nm – 475 nm, i.e., in the same range where the second peak emerged in the experiment with Au nanoparticle. Clearly, it is absent in this control experiment. The step seen at 500 s (directly upon closing of the batch reactor) in the time traces in both d) and e) are due to slight defocusing of the stage. f) Spectral position of the main negative RSID-peak at 520 nm that corresponds to the fluoresceine absorption band in alkaline conditions over time. We note that it, in contrast to the corresponding analysis in the presence of the Au nanoparticle depicted in* ***Figure 5f*** *in the main text, essentially stays constant.*


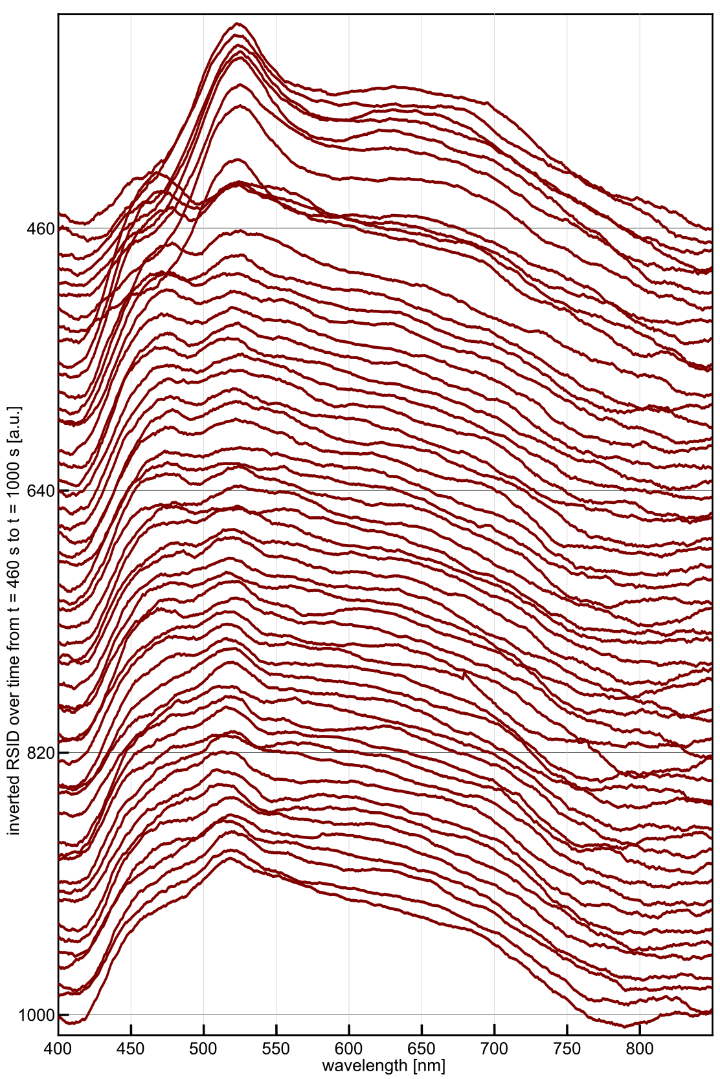


**Figure S4. *Ridge plot of NSS inverted RISD-spectra for section A of the nanochannel analyzed in Figure 5 in the main text.*** *The spectra plotted start at t = 460 s (reactant solution is flowing through the nanochannel) and are recorded up to 1000 s (batch reactor has been closed and working for 480 s).*


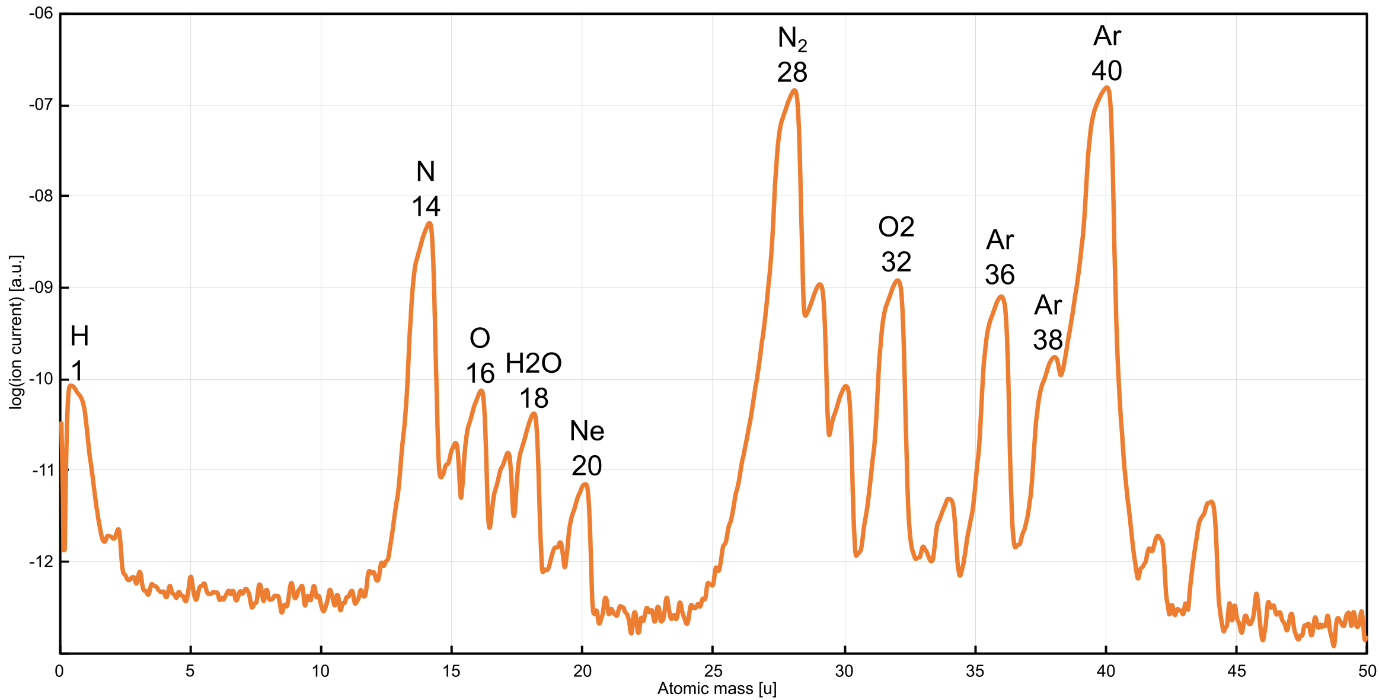


**Figure S5. *Mass spectrum of the N_2_ gas used in the batch reactor experiments.*** *The spectrum was taken with a Pfeiffer OmniStar GSD 320 gas mass spectrometer. Apart from the monoatomic (14 u) and diatomic (28) nitrogen fragments, there are various other gases present in the steam. Most prominent among them is Argon and its isotopes (36 u, 38 u, 40 u), but there are also traces of hydrogen (1 u) H_2_O (18 u) and Neon (20 u). In addition, and most importantly here, we also measure distinct signals that correspond to the monoatomic (16 u) and diatomic (32 u) fragments of oxygen, which corroborate the presence of sizable amounts of O_2_ in the N_2_ gas stream.*

**References**

[1] B. Altenburger, J. Fritzsche, C. Langhammer, *ACS Nano* **2025**, *19*, 2857.
